# EPCR in Wound Healing: Mechanisms of Action and Therapeutic Potential

**DOI:** 10.3390/cells15060567

**Published:** 2026-03-22

**Authors:** Hui Wang, Lyn March, Christopher J. Jackson, Marita Cross, Meilang Xue

**Affiliations:** 1 Sutton Arthritis Research Laboratory, Sydney Musculoskeletal Health, Kolling Institute, Faculty of Medicine and Health, The University of Sydney, Sydney, NSW 2065, Australia; 2 The Australian Arthritis and Autoimmune Biobank Collaborative (A3BC), Sydney Musculoskeletal Health, Kolling Institute, Faculty of Medicine and Health, The University of Sydney, Sydney, NSW 2065, Australia

**Keywords:** endothelial protein C receptor, wound healing, haemostasis, inflammation, proliferation, remodelling, protein C/activated protein C

## Abstract

The endothelial protein C receptor (EPCR) is an important component of the protein C (PC) system, recognised for its diverse roles in blood coagulation, inflammation, and stem cell regulation. Wound healing is a complex physiological process that can be divided into four distinct but overlapping phases: haemostasis, inflammation, proliferation and remodelling. Recently, EPCR has emerged as a key regulator in wound repair and regeneration. During haemostasis, EPCR enhances the conversion of PC to its activated form (APC) to optimise local and systemic anticoagulation. In the inflammatory phase, EPCR modulates immune cell activity, inhibits inflammatory factors, and maintains tissue barrier integrity. As the process transitions to the proliferative phase, EPCR promotes endothelial and epithelial cell proliferation, migration, neovascularisation and re-epithelization, and mediates the expression of matrix metalloproteinases to facilitate tissue reconstruction. Finally, during the remodelling phase, EPCR exerts a potential antifibrotic effect by regulating fibroblast activation and collagen deposition via the Transforming growth factor (TGF)-β1/Smad3 pathway, ensuring functional repair. While therapeutic potential has been shown in animal models, translating EPCR-mediated therapies to clinical application faces many challenges, including wound heterogeneity, dosage control, targeted delivery, and potential bleeding risks. Studies have shown that local drug delivery strategies, non-anticoagulant APC variants, and individualised treatment based on EPCR expression will be the key directions for future development. Additionally, EPCR may serve as a potential biomarker for assessing wound severity and guiding personalised interventions.

## 1. Introduction

The EPCR (CD201) is a type I transmembrane glycoprotein belonging to the MHC class I/CD1 family. Originally identified on the endothelial surface as a cofactor that enhances the activation efficiency of protein C (PC), EPCR has now been found to be expressed by multiple cell types, including keratinocytes, fibroblast lineages, dendritic cells, and fascial progenitors [1]. It is recognised as a multifunctional receptor with multiple ligands that mediates anticoagulation, inflammation, cytoprotection, vascular homeostasis, and stem cell biology [2].

Wound healing is a highly dynamic and tightly coordinated regenerative process in which the body responds to tissue injury [3]. Wound repair comprises four main phases: haemostasis, inflammation, proliferation, and remodelling [3,4]. Disruption of any stage may result in chronic non-healing wounds, hypertrophic scarring, or fibrotic conditions [3,5,6]. Chronic ulcers and hypertrophic scars are clinical challenges for which conventional treatments have limited efficacy [5,6]. It is estimated that approximately 1–2% of the global population is affected by chronic wounds at some point in their lifetime [4]. Conversely, affecting up to 70% of patients following deep thermal injury, hypertrophic scars remain within the boundaries of the original wound but result in significant functional impairment through contractures and mechanical tension abnormalities [7,8]. Despite advances in clinical treatments, the healing rate of chronic wounds remains unsatisfactory, highlighting the need for mechanistic research into the cellular and molecular regulators of wound repair, as well as the development of novel therapeutic agents [3].

Growing evidence indicates EPCR as a multi-functional regulator orchestrating haemostasis, inflammation, angiogenesis, epithelial repair, and fibrosis [1]. Its classical anticoagulant function provides the hemostatic foundation for early wound responses [9]. When bound to PC, EPCR not only accelerates the conversion of PC into an activated form (APC) but also acts as an essential cofactor for APC-mediated biased protease-activated receptor (PAR) 1 signalling [10,11,12]. This selective signalling triggers cytoprotective pathways, including anti-inflammation, anti-apoptotic, and barrier-stabilising responses, thereby actively participating in the inflammatory and proliferative stage [13,14,15]. During remodelling, EPCR suppresses Transforming growth factor (TGF)-β1/Smad3 fibrotic activation and contributes to structural resolution and functional repair [16]. The identification of EPCR as a marker for endothelial progenitors, keratinocyte stem cells, and fascial progenitor cells further emphasises its role as a multicellular coordinator capable of synchronising repair across vascular, epithelial and stromal compartments [17,18,19].

In this narrative review, we integrate current evidence on EPCR-mediated regulation across the four phases of wound healing, The literature discussed in this article was identified through searches of PubMed using key words including “EPCR”, “PC/APC”, and “wound healing”. By dissecting molecular pathways, cell-specific actions, and biased signalling mechanisms, we highlight EPCR as a regulatory hub that governs multicellular and multistage responses involved in repair. Finally, we discuss the translational potential and challenges associated with therapies targeting EPCR. This review also proposes precision medicine strategies that may guide future developments in wound repair.

## 2. EPCR and Its Regulation

### 2.1. Molecular Structure

EPCR is a glycoprotein receptor localised on the surface of various cells such as vascular endothelial cells, keratinocytes, fibroblasts and immune cells. Structural analysis by Oganesyan’s team demonstrated that the extracellular domain of EPCR comprises two α-helices and eight β-strands forming a hydrophobic lipid-binding groove, highly reminiscent of the antigen-presenting molecule CD1d [20]. EPCR is enriched within membrane lipid rafts (caveolae), a localisation that facilitates high-affinity interactions with its circulating ligands. Besides surface expression, EPCR also undergoes ligand-induced endocytosis, during which binding of APC or factor (F)VIIa triggers internalisation into early endosomes through a caveolin-dependent pathway [21]. Subsequently, EPCR traffics to Ras-related protein in brain (RAB11+) recycling endosomes and is returned to the cell surface [22]. This dynamic cycle maintains EPCR homeostasis, supports repeated ligand engagement, and highlights EPCR’s active role in cellular signal modulation.

### 2.2. Skin Distribution and Cell-Specific Expression

EPCR is mainly distributed in the endothelial cells of large blood vessels and in the skin. It is predominantly expressed in the dermal vascular endothelium and epidermal basal keratinocytes [18,23]. The expression pattern of EPCR in the skin can be categorised into constitutive and inducible modes. Constitutive expression is found in dermal microvascular endothelial cells, which exhibit the highest basal EPCR levels [24]. During wound healing, EPCR is expressed in keratinocytes at the wound edge [13], in stem/progenitor populations within the hair follicle and marks a critical population of fascia progenitors involved in wound closure [18,19]. EPCR is also highly expressed during embryonic skin development but progressively declines after birth, demonstrating its spatiotemporally dynamic regulatory pattern during development [18].

### 2.3. Regulatory Mechanism

Rather than remaining steady, EPCR expression and activity are dynamically regulated by both ligand engagement and microenvironment cues, enabling cells to adapt their signalling output during development and under stress conditions such as hypoxia, inflammation, and ischaemia [25].

In peripheral ischaemia, EPCR expression is selectively induced in endothelial cells exposed to reduced perfusion [26]. Bochenek and colleagues observed a progressive elevation of EPCR levels on the ischaemic side, with maximal expression detected at intermediate stages following injury [26]. Importantly, transcriptomic analyses revealed that disruption of PAR1 signalling altered clusters of genes involved in vascular homeostasis, inflammatory regulation, and nitric oxide biosynthesis [26]. These findings suggest the existence of a reciprocal regulatory loop, in which EPCR and its downstream effector, PAR1, mutually reinforce each other to stabilise endothelial transcriptional programs under stress.

Beyond transcriptional control, EPCR availability is also modulated at the post-translational level. Endothelial cells release sEPCR through regulated shedding, generating a circulating decoy receptor that sequesters PC and APC [27]. By adjusting local ligand availability, sEPCR indirectly shapes downstream signalling intensity, particularly pathways associated with nuclear factor (NF)-κB suppression and barrier maintenance [28]. This additional layer of regulation allows to fine-tune EPCR-mediated responses in a context-dependent manner [29].

EPCR regulation emerges from the integration of environmental stress signals, ligand availability, and receptor occupancy status. Through coordinated control of NF-κB activity, angiopoietin balance, and nitric oxide-related pathways, EPCR establishes a flexible regulatory framework that shapes cell behaviour during development and in pathological settings.

### 2.4. Functional Properties

EPCR shares a conserved α1–α2 domain architecture with the MHC class I/CD1 superfamily [30,31]. However, unlike these proteins, EPCR does not associate with β2-microglobulin and does not participate in antigen presentation [32,33]. Instead, this conserved structural platform enables EPCR to accommodate lipid-modified ligands and protease complexes, including PC, APC, and FVIIa [32,34]. This unique adaptation underpins EPCR’s function as a specialised signalling receptor rather than an immunological antigen-presenting molecule, allowing it to orchestrate ligand-dependent cytoprotective and reparative signalling across multiple tissues [20].

When EPCR is occupied by its physiological ligand, it not only serves as a scaffold for PC activation but also actively reprograms endothelial cell transcription and signalling outputs [35]. Joyce et al. demonstrated that APC binding to EPCR initiates extensive transcriptional reprogramming in endothelial cells, characterised by diminished NF-κB activity through reduced p50/p52 binding at promoter regions [35]. This shift leads to the downregulation of adhesion molecules, including Intercellular Adhesion Molecule 1 (ICAM-1), Vascular Cell Adhesion Molecule 1 (VCAM-1), and E-selectin, thereby limiting leukocyte recruitment and inflammatory amplification [35]. These findings indicate that EPCR functions as a molecular regulator, converting protease signalling from a damaging state to a protective state in a site-dependent manner.

#### 2.4.1. Ligand-Dependent Functions

**Figure 1:**
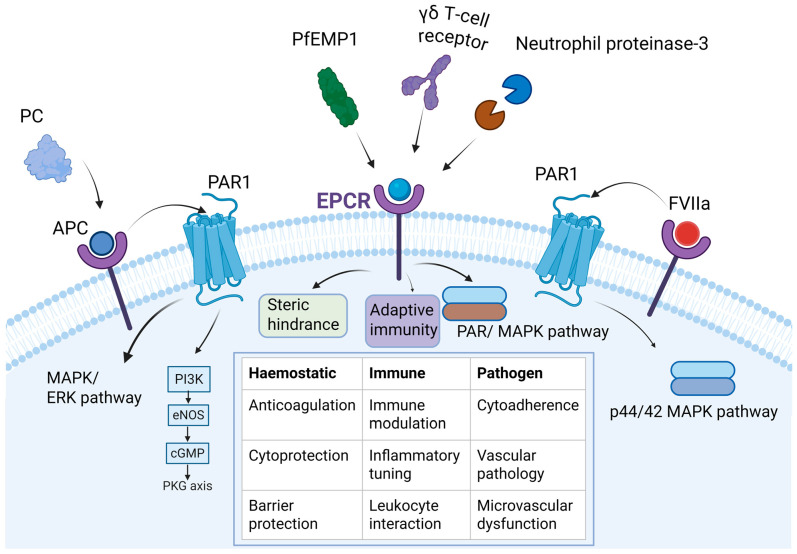
Endothelial protein C receptor as a multi-ligand signalling platform. PC: Protein C; APC: Activated protein C; PAR1: Protease-activated receptor 1; EPCR: Endothelial protein C receptor; PfEMP1: Plasmodium falciparum erythrocyte membrane protein 1; FVIIa: Activated factor VII; MAPK: Mitogen-activated protein kinase; ERK: Extracellular signal-regulated kinase; PI3K: Phosphoinositide 3-kinase; eNOS: Endothelial nitric oxide synthase; cGMP: Cyclic guanosine monophosphate; PKG: Protein kinase G.

The hydrophobic groove formed by EPCR accommodates a diverse array of lipid-modified ligands. Extensive research also demonstrates that EPCR functions as a multifunctional signalling platform, capable of being activated by multiple distinct ligands. Its primary ligands are PC or APC, which bind via hydrophobic residues within the Gla domain, driving anticoagulant and cell-protective signalling pathways [20]. Beyond the PC/APC pathway, EPCR further functions as a multifaceted platform by interacting with coagulation factor VIIa, thereby triggering barrier-protective PAR1 signalling [34,36]. Furthermore, EPCR engages with immune and pathogen-associated molecules, including neutrophil proteinase-3, γδ T-cell receptors, and the malaria parasite ligand PfEMP1 [37,38,39]. This diverse interaction network demonstrates EPCR’s function as a multi-ligand sensor, integrating haemostatic, inflammatory, and cytoprotective responses essential for wound repair (Figure 1).

- Binding PC/APC

The activation of PC to APC by PC-EPCR binding is regulated primarily by hydrophobic residues within the Gla domain of PC (e.g., Phe4 and Leu8) [20]. This interaction immobilises PC on the endothelial surface, enabling efficient activation by the thrombomodulin-thrombin complex [20]. Stearns-Kurosawa et al. further revealed that EPCR enhances activation efficiency through a “substrate entrapment” mechanism, which reduces the Km of PC activation and significantly boosts localised generation of APC, strengthening the anti-coagulant and cytoprotective capacity of the PC pathway [9].

**Figure 2:**
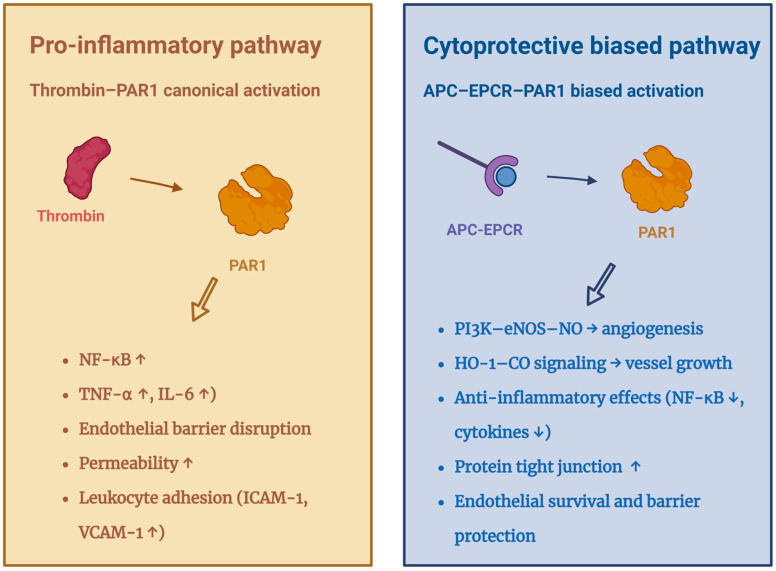
EPCR–APC signalling network and biased PAR1 activation. ↑: Increase; ↓: Decrease; APC: Activated protein C; EPCR: Endothelial protein C receptor; PAR1: Protease-activated receptor 1; PI3K: Phosphoinositide 3-kinase; eNOS: Endothelial nitric oxide synthase; NO: Nitric oxide; HO-1: Heme oxygenase 1; CO: Carbon monoxide; NF-κB: Nuclear factor kappa B; TNF-α: Tumour necrosis factor alpha; IL-6: Interleukin 6; ICAM-1: Intercellular adhesion molecule-1; VCAM-1: Vascular cell adhesion molecule-1.

In addition to PC activation, EPCR is indispensable for APC-mediated cytoprotective signalling (Figure 2). APC binds EPCR before PAR1 engagement, enabling biased activation of PAR1-Arg46, which stimulates MAPK pathways supporting anti-inflammatory, anti-apoptotic, and pro-survival gene programmes, according to the study of Riewald’s team [40,41]. Complementary findings by Uchiba et al. demonstrated that EPCR-APC signalling also activated the PI3K-eNOS-cGMP-PKG axis, promoting endothelial proliferation, survival, and in vivo angiogenesis [42,43].

- Interaction with other ligands

In addition to PC/APC, EPCR also interacts with molecules such as coagulation FVIIa [34], the malaria parasite ligand PfEMP1 [37], γδT-cell receptors [38], and neutrophil protease-3 [20]. This diverse interactome implies roles extending beyond haemostasis into immune modulation, pathogen sequestration, and lipid-associated inflammatory disorders [20]. Its upregulated expression in inflammatory and tumour microenvironments suggests a broader function in coordinating local coagulation, immune tone, and vascular regeneration, indicating EPCR as a flexible regulator across pathological states [36,44]. In endothelial cells, FVIIa binds directly to EPCR, activates endogenous PAR1, triggers p44/42 MAPK phosphorylation, and confers barrier protective effects, including suppression of thrombin-induced stress fibre formation and vascular leakage [45,46].

- EPCR cellular occupancy signal regulation dependency

Instead of acting as a stationary receptor, EPCR functions as a dynamic molecular switch, with the identity of its bound ligand determining the biological outcomes that follow [47]. Structural analysis reveals that the extracellular domain of EPCR contains a conserved hydrophobic α1–α2 groove designed to bind phospholipids, establishing the structural basis for its ligand-dependent functional reprogramming [32].

Under physiological conditions, this groove is constitutively occupied by endogenous phospholipids (primarily phosphatidylcholine), thereby maintaining the receptor in the conformation required for binding PC with high affinity [47]. Structural studies have demonstrated that phosphatidylcholine is the predominant lipid occupying this hydrophobic groove and contributes to maintaining the structural integrity required for efficient EPCR–PC/APC interactions [47]. However, this lipid occupancy can undergo pathological displacement. López-Sagaseta et al. demonstrated that bioactive lipids generated during inflammation, such as lysophosphatidylcholine or platelet-activating factor (PAF), can displace endogenous phospholipid loading [31]. This lipid exchange has been shown to impair EPCR-mediated PC/APC binding and downstream cytoprotective signalling [31]. 

Thus, specific lipid loading determines the signalling bias of the EPCR-PAR1 axis. While physiological lipid occupancy states support APC-mediated cell protection and barrier stability, replacement of these lipids by inflammatory derivatives disrupts this protective output [48]. Such ligand-dependent lipid occupancy therefore represents an additional regulatory layer controlling EPCR–APC signalling during inflammatory responses [48].

Beyond lipid exchange, EPCR function is further reprogrammed by other protein ligands. While APC binding triggers cell-protective pathways, factor VIIa binding induces distinct barrier-protective signalling via PAR1 [49]. Conversely, pathogen-derived ligands like the malaria protein PfEMP1 bind EPCR to sequester parasites while blocking the host’s protective APC signalling [37]. Moreover, EPCR interacts with γδ T cell receptors, linking vascular stress monitoring to adaptive immunity [44] (Figure 1).

These mechanisms position EPCR as a multi-ligand sensor. Its signalling function is continuously reprogrammed by specific combinations of lipid and protease ligands present in the microenvironment, enabling cells to switch between hemostatic, inflammatory, and immune response states.

#### 2.4.2. Stem Cell Regulation

Beyond its regulatory role in anticoagulation and inflammation, EPCR has emerged as an overarching signalling node for stem cell identity, self-renewal, and regenerative potential.

**Human epidermal keratinocytes:** EPCR has been identified as a key marker of epidermal keratinocyte stem cells (KSCs), where it colocalises with established stemness markers such as p63 and β1-integrin [50,51]. EPCR-high KSCs display enhanced colony-forming efficiency, strong holoclone-forming capacity, and sustained long-term self-renewal, together with robust epidermal regenerative potential [51,52]. Loss or blockade of EPCR causes apoptosis, reduced p63 levels, and pronounced impairment of proliferative and regenerative capacity in KSCs [51,52]. Mechanistically, EPCR maintains KSCs’ stemness through the APC-EPCR-PAR1-ERK axis, thereby supporting epidermal regeneration and re-epithelialisation during wound healing [51].

**Vascular endothelial stem/progenitor cells (VESCs):** EPCR is a core regulator for VESCs during both developmental and adult stages [53]. In human endothelial colony-forming cells (ECFCs), EPCR expression exceeds 95%, significantly surpassing that of classical progenitor markers such as CD34 and CD157 [54]. Functionally, EPCR maintains regenerative capacity by promoting cell cycle progression, clonogenicity, migration, and tubulogenesis, whereas EPCR deficiency induces G1 arrest and activates the TGF-β2/SMAD2/3 signalling axis [54]. Consistent with Yu et al.’s original identification, lineage tracing studies demonstrate that EPCR+ endothelial progenitor cells constitute the foundational vascular stem cell pool from embryonic day 7.5 onwards, participating in the formation of the yolk sac, placenta, and embryonic vascular networks [17,53]. Within adult tissues, EPCR+ endothelial cells sustain vascular growth over extended periods through clonal expansion, demonstrating their long-term regenerative capacity and contribution to vascular homeostasis [55,56]. Genetic knockout of EPCR+ cells results in severe vascular morphogenesis defects and embryonic lethality, underscoring the indispensable role of EPCR during development [57].

Fascial progenitor cells are specialized stem cells residing within the connective tissue (fascia) that act as a crucial, regenerative niche for tissue repair, particularly in wound healing and fibrosis. EPCR+ fascial progenitor cells form a critical regenerative node during wound healing [19]. These cells sequentially differentiate into proinflammatory fibroblasts, matrix-producing fibroblasts, and myofibroblasts, coordinating the inflammatory, proliferative and remodelling phases with precise temporal order [19]. Disruption of EPCR+ progenitor differentiation delays wound closure and disturbs the coordinated progression of repair phases, indicating that EPCR marks not only a lineage but also a functional executor of tissue remodelling programs.

**Hematopoietic stem cells (HSCs):** EPCR explicitly marks HSCs within the bone marrow [58]. As shown by Balazs’ team, EPCR+ cells display long-term multipotent reconstitution competence comparable to canonical standard HSC purification strategies and consistently localise within the side population trait pattern enriched for continuously propagating stem cells [59]. Importantly, EPCR expression closely aligns with classical HSC markers (Sca-1+, c-Kit+, CD34−) and defines a cellular subset with the highest efflux capacity, emphasising its mechanistic relevance in upholding stemness and bone-marrow repopulation strength [59]. Furthermore, its expression correlates with CXCL12-dependent retention, integrin α4β1 affinity, and spatial positioning near specific vascular microenvironments [60].

**Cancer cells:** EPCR functions as a determinant of stemness and metabolic reprogramming in multiple oncological settings [61]. As a crucial factor with various roles, EPCR is prominently illustrated in cancer models. For instance, in nasopharyngeal carcinoma, EPCR+ cells exhibit enhanced self-renewal, tumour-initiating capacity, metastatic dissemination, and chemoresistance. Mechanistically, EPCR overexpression drives lipid metabolic activation, lipid droplet accumulation, and mitochondrial remodelling, even in the absence of APC stimulation [62]. In addition, in aggressive triple-negative breast cancer cells, EPCR expression is associated with populations resembling cancer stem cells [58,61]. These populations exhibit tumour-initiating properties when examined in vivo [61].

In summary, EPCR functions not merely as a receptor within the PC anticoagulant pathway but as a multifaceted signalling centre governing coagulation balance, barrier protection, immune regulation, angiogenesis and stemness. Its unique structure and selective interaction with APC and phospholipids confer special capabilities that position EPCR as a central determinant of vascular and tissue homeostasis.

## 3. EPCR in Wound Healing

**Figure 3:**
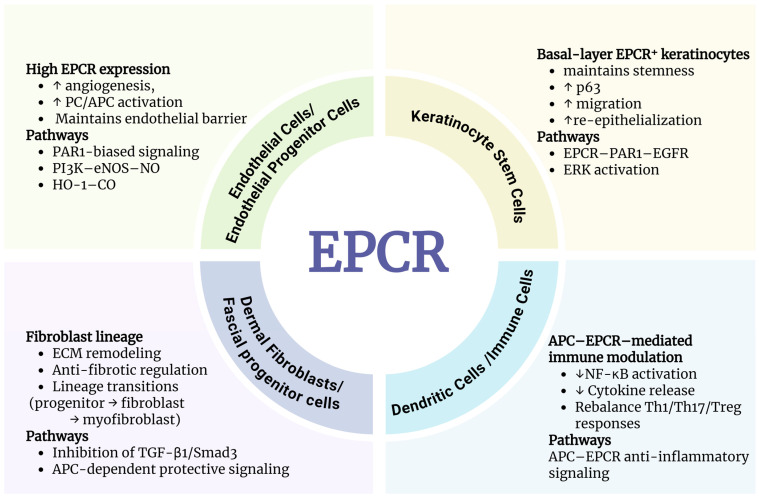
Cell-specific actions of EPCR in wound healing. EPCR: Endothelial protein C receptor; PC: Protein C; APC: Activated protein C; PI3K: Phosphoinositide 3-kinase; eNOS: Endothelial nitric oxide synthase; NO: Nitric oxide; HO-1: Heme oxygenase-1; CO: Carbon monoxide; ERK: Extracellular signal-regulated kinase; EGFR: Epidermal growth factor receptor; ECM: Extracellular matrix; TGF-β1: Transforming growth factor beta-1; Smad3: SMAD family member 3; NF-κB: Nuclear factor kappa B; Th1: T helper 1; Th17: T helper 17; Treg: Regulatory T cells.

Emerging evidence reveals that EPCR operates as a multifaceted regulator across the entire wound repair continuum (Figure 3). This section examines the specific roles of EPCR in four phases of wound healing, detailing its transition from a vascular stabiliser and immune modulator in early stages of injury to a critical driver of angiogenesis, epithelial regeneration, and anti-fibrotic remodelling in later stages.

### 3.1. The Coagulation Phase

Haemostasis and coagulation are key responses rapidly activated at the onset of wound healing, and EPCR plays a dual regulatory role at this stage. EPCR regulates haemostasis through three layers of control, which are biochemical enhancement of PC activation, early deployment of cytoprotective anti-inflammatory signalling, and developmental establishment of vascular integrity required for functional coagulation. EPCR regulates local coagulation by enhancing controlled anticoagulation while preventing excessive thrombin generation, primarily through increasing the efficiency of PC activation on the endothelial surface [63,64]. By localising PC to the endothelial surface, EPCR lowers its Km for the thrombin-thrombomodulin complex. This kinetic shift allows for rapid anticoagulant activation that complements, rather than disrupts, the initial clotting process [9]. On the other hand, by constructing a 5A-APC mutant, Mosnier et al. found that although this mutant lacks effective factor Va inactivation, it can still trigger cytoprotective signalling via the EPCR-PAR1 axis [14,65]. This indicates that during the coagulation phase, EPCR serves not only as a classical regulator of PC activation, but also as an early initiator of cytoprotective signalling essential for stable clot formation and prevention of microvascular injury. Additionally, Xue et al. showed that EPCR upregulation represents an intrinsic protective response following tissue injury [13]. Its interaction with 3K3A-APC, a mutant with reduced anticoagulant activity but preserved cytoprotective signalling, reduces immune cell infiltration and cytokine production while improving microcirculation. EPCR-deficient mice display exaggerated inflammatory responses and delayed repair, indicating EPCR’s indispensable role in early haemostatic microenvironment stabilisation [13]. From a development perspective, although EPCR-deficient mice do not show classical coagulation abnormalities, genetic studies reveal that EPCR+ cells are required for establishing embryonic vascular networks that provide the structural platform for haemostasis [53]. Thus, EPCR functionally acts as both a biochemical regulator of PC activation and a structural prerequisite for haemostatic competence.

### 3.2. The Inflammatory Phase

Inflammation is an essential stage of wound healing, but excessive or prolonged inflammation leads to impaired repair [66]. EPCR may serve as a key molecular “brake” that prevents excessive inflammation through multiple mechanisms, including inhibition of the NF-κB pathway, reduction in leukocyte adhesion and infiltration, and induction of anti-inflammatory cytokines [35,67]. This can ensure that the inflammatory phase proceeds efficiently while preventing pathological escalation.

In a murine allergic contact dermatitis model, Xue et al. showed that EPCR deletion dramatically exacerbates inflammation, as indicated by increased infiltrating immune cells, elevated levels of IL-6, TNF-α, IFN-γ, TGF-β1, and higher IgE levels [13]. This demonstrates that EPCR can help balance immune response under inflammatory stress. Further analysis showed that EPCR deficiency disrupts the inflammatory Th1/Th17/Treg equilibrium and increases activation of dendritic cells and mast cells, highlighting its role in controlling both innate and adaptive immunity [13].

Furthermore 3K3A-APC was shown to suppress cytokine production, modulate T-cell ratios, and reduce immune cell infiltration in an EPCR-dependent (or partially EPCR-independent) manner [24,65]. This supports the concept that EPCR-dependent APC signalling functions as a master regulator of inflammatory resolution rather than merely an anticoagulant modulator.

These findings position EPCR as a central anti-inflammatory molecule by regulating immune cell activation, cytokine release, and epidermal barrier function, and its lack of function significantly exacerbates the inflammatory response of the skin, suggesting that it is a potential target for regulating the treatment of chronic inflammation.

While EPCR plays an anti-inflammatory role in wound healing, its function may be tissue specific. For instance, in rheumatoid arthritis (RA), EPCR serves as a key driver for synovial inflammation. The functional outcomes of EPCR signaling appear to depend largely on the specific conditions. EPCR can interact with a variety of ligands, including APC, coagulation factors, antiphospholipid antibodies, and inflammatory lipid mediators, which activate different downstream signaling pathways depending on the cell and inflammatory environment [68]. Studies on RA have shown that EPCR is expressed in both synovial tissue and immune cells and can modulate inflammatory responses as well as the behavior of synovial fibroblasts [68]. These observations suggest that EPCR may exert either an anti-inflammatory or pro-inflammatory effect, depending on the tissue environment and ligand interactions (also see Section 3.3.4). This contrasts sharply with physiological wound repair, where EPCR helps to ensure the timely resolution of inflammation.

### 3.3. The Proliferative Phase

#### 3.3.1. EPCR in Vascular Expansion and Angiogenic Support

During the proliferative phase, restoration of tissue perfusion and microvascular networks is a prerequisite for subsequent tissue reconstruction [3,69]. Increasing evidence indicates that EPCR functions as a central regulator of angiogenic responses under regenerative conditions [26,46].

Bochenek et al. demonstrated that EPCR expression is markedly upregulated in endothelial cells within ischaemic tissues, peaking at days 7 and 14 post-injury, and that endothelial-specific EPCR deficiency results in impaired neovascularisation and delayed perfusion recovery [26].

Mechanistically, EPCR-dependent biased PAR1 signalling sustains endothelial nitric oxide (NO) bioavailability, as APC cleaves PAR1 at Arg46 rather than the canonical thrombin site Arg41, thereby activating cytoprotective and pro-angiogenic pathways [26,65].

In a complementary pathway, elevated EPCR expression within pathological vascular plexuses induces heme oxygenase-1 (HO-1) and carbon monoxide (CO) production, and administration of CO donors partially rescues angiogenic defects in EPCR-deficient settings, establishing the EPCR–HO-1–CO axis as an additional pro-angiogenic mechanism [46,70].

Beyond mature endothelium, EPCR supports vascular expansion by functionally sustaining a regenerative endothelial progenitor pool rather than redefining its identity [17,54]. Lineage-tracing and genetic ablation studies demonstrate that Procr^+^ progenitors provide the cellular reservoir required for vascular establishment, and that their loss leads to catastrophic vascularisation failure [53,57]. At the mechanistic level, EPCR maintains progenitor competence by promoting cell cycle progression and suppressing the antiproliferative TGF-β2/SMAD2/3 axis, thereby preserving clonogenic capacity and high proliferative potential during vascular reconstruction [23,42].

#### 3.3.2. EPCR-Mediated Epithelial Reconstruction and Matrix Remodelling

In parallel with vascular expansion, epithelial restoration and extracellular matrix remodelling represent essential components of proliferative tissue repair. EPCR contributes to these processes by regulating keratinocyte behaviour and matrix dynamics.

In keratinocytes, EPCR expression is inducible and functionally responsive [13]. APC upregulates EPCR and PAR1 expression, stimulates proliferation, activates ERK/p38 signalling, and enhances MMP-2 production, effects fully abolished when EPCR or PAR1 is blocked [13]. This indicates that EPCR abundance mirrors local regenerative demand, making it a potential indicator of epithelial activation and re-epithelialisation capacity.

Jackson and Xue et al. demonstrated that APC induces matrix metalloproteinase-2 (MMP-2) expression through an EPCR-dependent mechanism, promoting endothelial and keratinocyte migration while selectively suppressing inflammation-associated MMP-9 [71,72]. This differential regulation supports controlled extracellular matrix remodelling while limiting excessive inflammatory interference.

EPCR is also prominently expressed within the epidermal basal layer, where it colocalises with core keratinocyte stem cell markers, including p63 and integrin β1 [18]. It is particularly enriched in a subpopulation of small-sized keratinocytes with high clonogenic capacity and prolonged proliferative potential, indicating an association with epidermal stemness [18]. Functional blockade or downregulation of EPCR induces keratinocyte apoptosis, suppresses p63 expression, and reduces clonogenic efficiency, whereas EPCR overexpression enhances holoclone formation and 3D epidermal reconstruction [18].

Moreover, EPCR-mediated APC signalling accelerates keratinocyte migration and epithelial closure in vivo [71]. APC activates the EPCR-PAR1-EGFR-Tie2 signalling cascade, while upregulating junctional proteins such as ZO-1, Claudin-1, and VE-cadherin [15]. These effects facilitate the rapid re-establishment of epithelial barrier integrity following injury. In murine full-thickness skin wound models, local APC administration markedly enhanced re-epithelialisation rates and improved epidermal stratification, reinforcing the functional significance of the EPCR/APC axis in epithelial regeneration [15,71].

Altogether, during the proliferative phase, EPCR supports tissue reconstruction through coordinated regulation of vascular expansion, progenitor cell function, epithelial restoration, and matrix remodelling. By integrating biased PAR1 signalling, progenitor cell maintenance, and barrier reinforcement, EPCR orchestrates a regenerative microenvironment conducive to effective tissue rebuilding. These multi-level actions position EPCR as a central regulator of proliferative tissue repair rather than a passive anticoagulant receptor.

#### 3.3.3. The Remodelling Phase

The remodelling phase of wound healing, characterised by apoptotic clearance, matrix remodelling, collagen deposition, and the restoration of tissue tension, represents a critical stage for achieving functional repair [73]. During this phase, EPCR jointly shapes the reparative microenvironment through APC-dependent signal regulation and the participation of EPCR+ progenitor cells in tissue reconstruction, a dual mechanism [16,73].

At the signalling level, APC activates the PAR1 cascade via EPCR, exerting significant anti-fibrotic and tissue-protective effects. Research by Giri et al. demonstrated that in a chlorhexidine-induced peritoneal fibrosis model, APC significantly suppressed TGF-β1-induced Smad3 hyperphosphorylation, reduced type I/III collagen deposition, and inhibited the upregulation of mesenchymal phenotypic molecules such as α-SMA, vimentin, and N-cadherin, thereby limiting the formation of a fibrotic microenvironment [16]. Furthermore, the signal-biased variant APC-2Cys retained anti-fibrotic effects, whereas the anticoagulant-biased APC-E170A exhibited reduced activity, and the protease-inactivating mutation (APC-S195A) was completely ineffective, demonstrating that its tissue-protective action depends on EPCR-PAR1 signalling rather than anticoagulant function [65]. At the cellular level, APC also inhibits TGF-β1-induced expression of fibroblast activation-related genes (α-SMA, Fibronectin, Smad3), suggesting direct regulatory capacity over pathological matrix remodelling during the remodelling phase [74].

At the cellular origin and tissue repair orchestration level, EPCR participates in tissue remodelling as a key marker for both adult fascial progenitor cells and the mesenchymal repair cell pool [19]. Recent studies reveal that EPCR^+^ fascial progenitor cells undergo rapid activation and recruitment post-injury. Functioning as pivotal executors in local tissue repair, they orchestrate the wound microenvironment by responding to injury signals, coordinating the overall repair response, and contributing to the regenerative cell pool [19]. This establishes their foundational role in structural reconstruction during the remodelling phase [19]. These cells possess adult mesenchymal progenitor cell properties, maintaining tissue homeostasis and participating in post-injury repair and remodelling responses [75]. This indicates that the EPCR-marked progenitor cell system serves not only as a cellular source for injury response but also as a crucial coordinator of tissue tension and structural functional recovery of the tissue architecture [73].

Overall, during the remodelling phase of wound healing, EPCR drives tissue structural reconstruction and microenvironmental homeostasis restoration through dual pathways of ‘signal regulation + cellular engagement’. At the signalling level, EPCR-mediated APC-PAR1 preferential signalling suppresses excessive TGF-β1/Smad3 activation, thereby mitigating fibroblast phenotypic transformation and excessive collagen deposition to achieve anti-fibrotic and tissue-protective effects. At the cellular level, EPCR+ fascial and mesenchymal progenitor cells are rapidly mobilised post-injury, undertaking the coordinated roles of replenishing the wound cell pool, responding to repair signals, and remodelling tissue structure. As a result, EPCR serves not only as a signalling hub for inhibiting pathological remodelling but also as a pivotal coordinator for restoring tissue tension and structural reconstruction, providing a crucial biological theoretical foundation for limiting scarring and achieving functional repair.

#### 3.3.4. EPCR as a Potential Biomarker for Wound Healing

Accumulating evidence from mechanistic and translational studies suggests that EPCR is measurable and dynamic when responding to injury, which makes it a promising biomarker candidate across inflammatory, ischaemic, and wound repair. Several studies support this emerging direction. EPCR undergoes regulated ectodomain shedding mediated by ADAM17, producing soluble (s)EPCR, whose abundance increases under inflammatory stimulation and vascular stress [27]. This regulated shedding process releases the extracellular domain of EPCR into the circulation, allowing soluble EPCR to be detected in plasma under physiological and inflammatory conditions [27]. Because sEPCR competes with membrane EPCR for PC/APC binding [28], elevated circulating sEPCR levels reflect endothelial dysfunction, impaired anticoagulant capacity [76], and altered APC signalling, features that may be clinically meaningful in trauma, infection, or wound states [29]. sEPCR retains the ability to bind PC and APC, thereby modulating PC pathway activity in the circulation [28].

Independent clinical observations, such as those reported by Zhao et al., show that EPCR expression in burn wounds correlates with tissue injury severity and treatment intensity, suggesting predictive value for stratifying patients in high-inflammation or high-turnover reparative environments [77]. Incorporating EPCR abundance, APC/PC ratios, vascular leakage indicators, and inflammatory mediator profiles may therefore help define personalised therapeutic windows, estimate wound responsiveness, and optimise APC-based or regeneration-targeted strategies [76,77].

In summary, the current findings indicate that EPCR functions across multiple cells during wound healing. In endothelial cells, EPCR primarily regulates anticoagulant functions, vascular stability, and angiogenic responses; in keratinocytes, EPCR promotes epithelial regeneration and re-epithelialization through APC-related signaling; in the stromal lineage, EPCR-expressing fibroblasts or progenitor cell populations participate in extracellular matrix remodeling and tissue structural reconstruction; and in immune cells, EPCR signaling contributes to the regulation of inflammatory responses and the maintenance of immune homeostasis. Therefore, EPCR should not be viewed as a receptor confined to a single cell type in wound healing, but rather understood as a key regulator that synergistically modulates vascular, inflammatory, epithelial, and stromal repair processes across multiple cellular compartments.

## 4. Clinical Considerations and Challenges for EPCR-Targeted Wound Therapies

Although no clinical trials have yet directly targeted EPCR itself, accumulating translational evidence indicates that the EPCR–APC–PAR1 signalling axis already possesses clinically actionable properties that extend beyond experimental wound models. A principal challenge lies not in biological plausibility, but in translating EPCR-dependent cytoprotective signalling into interventions that are both effective and safe across heterogeneous clinical settings. In addition, most currently available clinical evidence derives from studies of APC, which exerts many of its cytoprotective and anti-inflammatory effects through binding to EPCR and subsequent activation of PAR1 signalling pathways.

### 4.1. EPCR Agonists

One major obstacle arises from the dual nature of APC [78] While endogenous APC exerts well-characterised cytoprotective, anti-inflammatory, and barrier-stabilising effects through EPCR-dependent signalling, its native anticoagulant activity restricts systemic therapeutic use due to bleeding risk [79]. The PROWESS randomized controlled trial evaluated recombinant APC (activated drotrecogin alfa) administered as an intravenous infusion at 24 μg/kg/h for 96 h, with 28-day all-cause mortality as the primary endpoint. Treatment significantly reduced mortality in patients with severe sepsis, although an increased risk of bleeding complications was observed [79]. In addition, whole-body administration can induce acute traumatic coagulopathy and destabilise circulatory homeostasis, particularly in patients with severe burns or polytrauma [80]. This limitation has been clearly illustrated in earlier sepsis trials and has driven the development of APC variants that preserve EPCR/PAR1-mediated signalling while attenuating anticoagulant potency [79,81]. APC variants with normal cytoprotective but reduced anticoagulant activity demonstrate that EPCR-dependent cytoprotective signalling can be pharmacologically uncoupled from bleeding risk [14]. This positions EPCR not only as a therapeutic co-factor but also a mechanistic marker reflecting the tissue’s ability to respond to APC-based interventions.

Beyond neurological indications, clinical observations in cutaneous and chronic wounds further support the therapeutic relevance of APC–EPCR signalling. Wijewardena, Whitmont and colleagues reported that local administration of APC in patients with severe, non-healing pressure ulcers and chronic diabetic lower leg ulcers resulted in rapid granulation tissue formation, enhanced angiogenesis, and complete epithelial closure, even after prolonged failure of conventional wound therapies [82,83]. Importantly, APC was delivered locally rather than systemically, and no bleeding complications were observed. These findings align with broader clinical and preclinical evidence summarised by Zhao et al., indicating that topical or locally confined APC delivery consistently promotes wound resolution while avoiding systemic adverse effects [84,85]. Collectively, these studies underscore that EPCR-dependent pathways can be therapeutically engaged in human tissues, even in the absence of direct EPCR-targeted agents.

### 4.2. Receptor Availability

A fundamental but often overlooked translational challenge is the dependency of EPCR signalling on its specific subcellular localisation. Beyond simple surface expression, functional EPCR signalling requires the receptor to be partitioned within cholesterol-rich membrane microdomains known as lipid rafts or caveolae.

Russo and colleagues provided definitive evidence that EPCR co-localises with Caveolin-1 in endothelial lipid rafts, a spatial organisation essential for efficient PC activation and cytoprotective signalling [86]. Disruption of these lipid rafts, common in pathological states involving membrane remodelling or cholesterol dysregulation, leads to the displacement of EPCR from signalling hubs, effectively silencing its protective output even when total protein levels remain unchanged [86]. Furthermore, oxidative stress, a hallmark of wound environments, triggers the phosphorylation of EPCR via PKC-dependent pathways, which promotes its translocation out of caveolae and accelerates its proteolytic shedding [27]. This implies that clinical interventions must not only maintain EPCR expression but also preserve membrane lipid integrity. Therapies that fail to stabilise these microdomains may face “signalling resistance,” representing a sophisticated pharmacological barrier to EPCR-targeted wound treatments.

Pathological heterogeneity profoundly influences the therapeutic responsiveness of the EPCR-APC axis. EPCR expression varies dynamically with wound depth, inflammatory load, microvascular collapse, and re-epithelialisation status. Such spatiotemporal heterogeneity implies that targeting EPCR using APC at the same dose in different circumstances is biologically unrealistic and may result in suboptimal or even paradoxical responses [87]. Therefore, precision dosing strategies should integrate EPCR expression profiling, local haemodynamic status, and inflammatory biomarkers [87].

Another translational consideration relates to receptor availability and regulation. EPCR expression is dynamically modulated by inflammatory stimuli and proteolytic shedding, particularly through ADAM17-mediated cleavage [27]. Loss of surface EPCR may blunt endogenous protective signalling, suggesting that therapeutic strategies aimed at preserving or restoring EPCR membrane expression—rather than solely supplying exogenous ligands—could enhance tissue resilience in inflammatory or ischaemic contexts. Although such approaches remain preclinical, they represent a conceptually distinct intervention point within the EPCR pathway.

### 4.3. Regenerative Stem Cell Populations

While EPCR is established as a marker for hematopoietic and endothelial progenitors, recent discoveries reveal its broader role as a universal “anchor” required for stem cell retention within diverse solid tissue niches. This function poses a unique challenge: protecting the physical interaction between stem cells and their microenvironment during therapy.

Groundbreaking research by Wang and colleagues identified EPCR as a distinct marker of multipotent mammary stem cells, where it does not merely mark the cell but actively regulates its stability within the basal niche [88]. In this context, EPCR interacts with the Wnt signalling pathway to maintain stemness; its genetic ablation or loss drives cells toward premature differentiation, thereby depleting the regenerative reservoir [88]. Similarly, within the bone marrow niche, the physical retention of stem cells is governed by an “occupancy” mechanism. Gur-Cohen et al. demonstrated that EPCR acts as a retention signal, and its cleavage by inflammatory proteases triggers the immediate egress of stem cells into circulation, effectively “evicting” them from their protective niche [60]. Therefore, a critical prerequisite for EPCR-based regenerative therapy is the maintenance of the “receptor-ligand tether.” Therapeutic strategies should be designed to prevent the inflammation-induced cleavage of this tether, ensuring that the progenitor pool remains physically anchored and biologically potent throughout the prolonged phases of tissue reconstruction.

## 5. Conclusions

**Figure 4:**
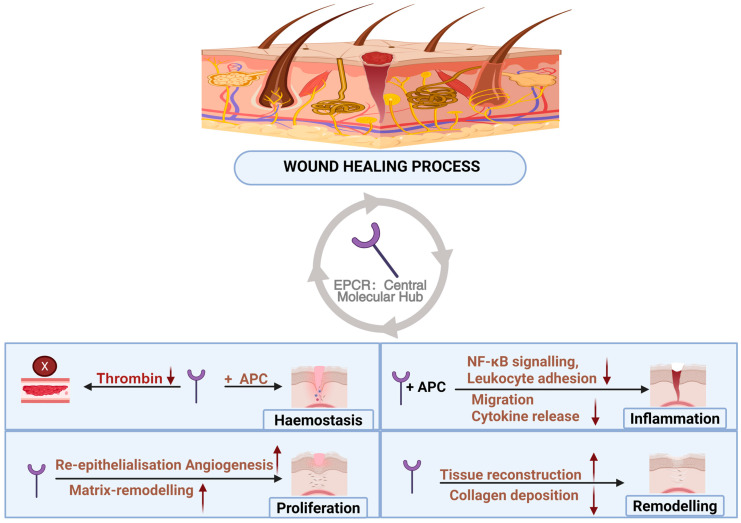
Overview of EPCR-mediated regulation across the phases of wound healing. EPCR: Endothelial protein C receptor; APC: Activated protein C; NF-κB: Nuclear factor kappa B.

Wound healing is an organised, multi-stage biological program that requires precise coordination among haemostasis, inflammation, angiogenesis, re-epithelialisation, and matrix remodelling (Figure 4). Recent advances have fundamentally reshaped the understanding of EPCR in wound healing, revealing that it functions far beyond its classical anticoagulant role. Instead, EPCR emerges as an integrative molecular hub that connects vascular stability, immune modulation, stem cell maintenance, and anti-fibrotic tissue regeneration. Mechanistically, the receptor regulates this continuum by converting early coagulation-inflammation stimuli into cytoprotective pathways, driving stem cell-mediated revascularisation and re-epithelialisation, and ultimately restricting fibrotic overgrowth via the suppression of TGF-β signalling.

Looking ahead, the EPCR-APC axis represents a promising therapeutic target for wound repair. The development of non-anticoagulant APC variants, EPCR-targeted biomaterials, and localised delivery platforms is expected to substantially enhance clinical safety. Moreover, the dynamic expression of EPCR suggests its potential as a biomarker for stratified therapy, enabling more precise and personalised interventions. As mechanistic studies deepen and translational research progresses, EPCR-centred therapeutic strategies may evolve into a clinically actionable paradigm for chronic wounds, traumatic injuries, and fibrotic disorders.

In summary, EPCR serves as a central integrator of inflammatory control, vascular regeneration, epithelial repair, and anti-fibrotic remodelling. Its unique cross-lineage regulatory capacity positions it as both a mechanistic cornerstone of wound biology and a strategically compelling target for next-generation regenerative medicine.

## Data Availability

No new data were created or analyzed in this study. Data sharing is not applicable to this article.
